# Multi-omics analysis revealing a senescence-relevant lncRNAs signature for the assessment of response to immunotherapy for breast cancer

**DOI:** 10.1097/MD.0000000000034287

**Published:** 2023-07-14

**Authors:** Ziyi Yu, Yanhui Zhu, Jie Ji

**Affiliations:** a Jiangsu Breast Disease Center, The First Affiliated Hospital of Nanjing Medical University, Nanjing, China.

**Keywords:** breast cancer, immunotherapy, lncRNA, multi-omics, senescence

## Abstract

Breast cancer (BRCA) is the most fatal malignancy of women. Immunotherapy has greatly improved the prognosis of advanced BRCA. Cellular senescence contributes to tumorigenesis and suppresses anti-cancer immunity. Identification of senescence-relevant long noncoding RNAs (SRlncRNAs) signature may benefit the predictions of prognosis and response to immunotherapy of BRCA. RNA-seq, mutation, and clinical data of BRCA were acquired from public databases. SRlncRNAs were screened using univariate Cox regression analysis. Consensus clustering classified BRCA patients into 2 clusters, and the differences of overall survival (OS) and immune status between the 2 clusters were analyzed by survival analysis, CIBERSORT, and ESITIMATE. The SRlncRNAs signature was constructed by least absolute shrinkage and selection operator (LASSO) regression analysis, and BRCA patients were divided into 2 risk groups. Enrichment analyses were performed to explore the cancer- and immunotherapy-relevant pathways. Transcriptome analysis was performed to investigate the differences of OS, immune infiltration, and ESITIMATE score of the 2 groups. Genome analysis was applied to investigate the differences of somatic mutation, tumor mutation burden (TMB) and microsatellite instability (MSI) between the 2 risk groups. A nomogram combined with calibration curves and decision curve analysis (DCA) was established for better clinical decision. Tumor Immune Dysfunction and Exclusion (TIDE) score and IMvigor-210 were applied for the predicting of response to immunotherapy. Profiling Relative Inhibition Simultaneously in Mixtures (PRISM) and the Cancer Therapeutics Response Portal resource (CTRP) databases were used for drug susceptibility analysis. Ten prognostic SRlncRNAs were identified and BRCA patients were divided into 2 clusters. Cluster 1 had better OS with anti-tumor immune microenvironment. The high-risk BRCA had poorer OS in the Cancer Genome Atlas (TCGA) training cohort, which was also verified by TCGA validation cohort and GSE20685 validation cohort. Low-risk patients also had anti-tumor immune microenvironment. Genome analysis demonstrated that the high-risk group had significant higher TMB. High-risk BRCA were more susceptive to immunotherapy according to the TIDE score and IMvigor-210. Finally, drug susceptibility analysis showed that 6 compounds were sensitive to high-risk BRCA patients. We developed and verified an original SRlncRNAs signature by multi-omics analysis, which could serve as a prognosis and immunotherapy predictor for BRCA.

## 1. Introduction

According to global cancer epidemiology data, breast cancer (BRCA) has already became the most prevalent and fatal malignancy of women due to its high heterogeneity.^[1]^ Every year, almost 2300,000 women are diagnosed as BRCA and 685,000 BRCA patients died.^[2]^ Thus, BRCA has contributed to a significant global cancer burden. Based on hormone receptors status and pathological stage, the treatment of BRCA includes surgery, chemotherapeutics, targeted treatment, hormone replacement therapy, immunotherapy.^[3]^ However, for advanced BRCA patients, the effectiveness of the above treatments remains poor due to insensitivity to chemotherapy, highly aggressive behavior, and loss of mutation targets.^[4]^

The crucial function of the immune system in the tumorigenesis of BRCA has been widely verified.^[5,6]^ Immunotherapy, mainly consisting of immune checkpoint inhibitors (ICIs) and immune cell therapy, has significantly improved the outcomes of advanced BRCA. The objective response rate to nivolumab can achieve 20% in metastatic triple-negative BRCA.^[7]^

Long noncoding RNAs (lncRNAs) are unable to translate proteins directly; instead, they regulate the transcriptional, translation and modification of genes, which has a significant impact on the development and progression of malignant tumors.^[8]^ Zhang et al found that lncRNA FOXD3-AS1 could promote tumor development and metastasis by miR-127-3p/ARF6 axis in BRCA.^[9]^ Xu et al found lncRNA Uc003xsl could activate an epigenetic-driven NFκB/IL8 cascade thus contribute to tumor metastasis and poor survival in triple-negative BRCA patients.^[10]^

Cellular senescence is a frontier hallmark of cancer.^[11]^ Senescent cells could contribute tumor malignization through enhancing proliferative signaling, reducing cell apoptosis, promoting angiogenesis and metastasis, and suppressing anti-cancer immunity.^[12–14]^ For BRCA, studies of cellular senescence have focused on the mRNA and protein levels. Chen et al found that upregulated ACLY can induce resistance to docetaxel, which is c9elated with worse tumor relapse-free survival in BRCA patients.^[15]^ Wu et al found that the overexpression of AKR1B1 can activate NF-κB, thus promoting tumorigenicity and metastasis of basal-like BRCA.^[16]^ However, few scientists have reported the effect of senescence-relevant lncRNAs (SRlncRNAs) in predicting clinical prognosis and efficacy of immunotherapy of BRCA.

In current study, we comprehensively explored the connection between SRlncRNAs and prognosis of BRCA at the transcriptomic and genomic levels. We construct and verify an original and dependable SRlncRNAs signature, and investigate its correlation with overall survival (OS), enrichment pathways, immune infiltration, immune checkpoint expression, genomic mutation, tumor mutation burden (TMB), microsatellite instability (MSI), drug susceptibility, and effectiveness of immunotherapy. Eventually, we developed a precise nomogram for preferable clinic decision.

## 2. Materials and methods

### 2.1. Transcriptomic and genomic data sources

The RNA-seq, mutation and survival data of BRCA were obtained from The Cancer Genome Atlas (TCGA) database. GSE20685 from Gene Expression Omnibus database included gene expression profiles and survival data of BRCA, which was used as external validation. The list of senescence-relevant genes is downloaded from CellAge (see Table S1, http://links.lww.com/MD/J310, Supplemental Content, which illustrates the list of senescence-relevant genes).^[17]^ R package “sva” and Perl software were applied for data normalization. Patients with incomplete clinical or gene expression information were excluded.

### 2.2. Screening of prognostic senescence-relevant genes and identification of SRlncRNAs expression data

Univariate Cox regression analysis was performed to screen prognostic senescence-relevant genes by R package “survival” (*P* < .05). LncRNAs were screened using the human reference genome website (GRh38.104). Then, Pearson correlation analysis was applied to screen SRlncRNAs (*P* < .001; |R| > 0.4) using the “cor” function of R. To ensure the reliability of our study, univariate Cox regression analysis was implemented once again to identify prognostic SRlncRNAs (*P* < .05). Eventually, we compared the expression levels of prognostic SRlncRNAs in the BRCA and normal tissue in TCGA database using R packages “pheatmap” and “ggplot2.”

### 2.3. Consensus clustering analysis of prognostic SRlncRNAs

Unsupervised clustering divided BRCA patients into certain subtypes with the help of “ConsensusClusterPlus” package. A total of 1069 patients were classified into 2 clusters. To decode the differences between 2 clusters, survival analysis and clinicopathologic features analysis were performed using R packages “survival” and “pheatmap.” Further, we investigated the different immune status between the 2 clusters, mainly including immune checkpoint analysis and immune infiltration analysis by CIBERSORT^[18]^ and ESITIMATE^[19]^ algorithm. In the ESITIMATE algorithm, StromalScore represents the level of infiltration of stromal cells and ImmuneScore represents the level of infiltration of immune cells. ESITIMATEScore is the sum of StromalScore and ImmuneScore. We used “CIBERSORT R script v1.03” and R package “estimate” to perform the CIBERSORT and ESITIMATE algorithm. R packages “ggplot2,” “corrplot,” and “vioplot” were applied for the visualization.

### 2.4. Establishment and validation of SRlncRNAs signature

Using the “glmnet” package, the least absolute shrinkage and selection operator (LASSO) regression analysis could restrict the SRlncRNAs which were most relevant to OS. The TCGA cohort was classified into training or internal validation cohort in the ratio of 1:1. GSE20685 cohort was used for external validation. The signature was established by calculating the risk score of patient: risk score = sum of LASSO regression coefficient × included SRlncRNA expression. Using the median as cut off, BRCA patients were classified into low- or high-risk group. With the help of “survival” package, we investigated the differences of OS between the 2 risk groups in the TCGA training cohort, the result was verified in the TCGA internal validation cohort and GSE20685 external validation cohort. Using R packages “timeROC” and “survminer,” we quantified the accuracy of the SRlncRNA signature by the area under the curve (AUC) of the 1-, 3-, 5-year receiver operating characteristic (ROC) curves. Using “survival” package, independent prognostic analyses were implemented to estimate whether SRlncRNA signature is an independent prognostic variable of BRCA in TCGA cohort and GSE20685 cohort.

### 2.5. Decoding the SRlncRNAs signature in aspect of tumor immunology using transcriptome analysis

Using “pheatmap” package, clinicopathologic features analysis was implemented to compare whether differences were existing in age, TNM stage, and pathological stage between the 2 risk groups. Different immune status of the 2 risk groups were explored just as Part 2.3. R package “limma” helped find the differentially expressed genes (DEGs) (*P* < .05). Gene Ontology (GO) and Kyoto Encyclopedia of Genes and Genomes (KEGG) analyses were conducted and visualized to reveal enrichment pathways with the help of R packages “clusterProfiler” and “ggplot2.” Gene set enrichment analysis (GSEA) was implemented to explore the pivotal tumor-relevant pathways of SRlncRNAs signature (FDR < 0.25), which was visualized by “ggplot2.”

### 2.6. Decoding the SRlncRNAs signature using genome analysis

TCGA-BRCA genomic mutation data (mutect2) was downloaded through R package “TCGAbiolinks.” Patients with incomplete mutation information were excluded. Package “maftools” was used to investigate and visualize the top 20 genes in the 2 risk groups in terms of mutation frequency. Then, Perl script was used to obtain the TMB and MSI of patients, and “ggplot2” made the visualization.

### 2.7. Nomogram, calibration curves and decision curve analysis (DCA) for clinical decision

The SRlncRNAs signature was integrated with other clinicopathological features to plot a nomogram for more convenient clinical use. One-, 3-, and 5-year calibration curves verified the its precision. Eventually, DCA was applied to verify the clinical benefit of the nomogram. R packages “regplot,” “rms,” “survival,” and “stdca.R.”

### 2.8. Response to immunotherapy and drug susceptibility

Tumor Immune Dysfunction and Exclusion (TIDE) score could be served as a predictor of response to immunotherapy in oncology patients, and a higher TIDE score means a lower response rate to ICIs and a poorer OS.^[20]^ We organized the gene expression matrix into a TIDE input file via R, and calculated TIDE score via the official website (http://tide.dfci.harvard.edu/). Finally, we compared the TIDE score of BRCA patients in the 2 risk groups using R package “ggplot2.” IMvigor-210 is an ICIs therapy cohort of bladder cancer with RNA-seq data.^[21]^ We constructed the SRlncRNAs signature in the IMvigor-210 cohort, and compared the difference in response rates to ICIs therapy between patients in the 2 risk groups. Eventually, we screened for drugs that were sensitive to high-risk BRCA patients using the Profiling Relative Inhibition Simultaneously in Mixtures (PRISM) (www.theprismlab.org/), the Cancer Therapeutics Response Portal resource (CTRP),^[22]^ and the Cancer Cell Line Encyclopedia project.^[23]^

### 2.9. Statistical analysis

R version 4.1.1 performed all the statistical analysis. *P* < .05 was the cut off. Student *t* test was applied to compare the expression of immune checkpoint, TMB, MSI, and TIDE score. Correlation analysis was implemented by Pearson correlation analysis. Chi-square test was implemented for response rates to ICIs. Survival analysis was conducted by log-rank test.

## 3. Result

### 3.1. Screening the prognostic SRlncRNAs in BRCA

**Figure 1.**
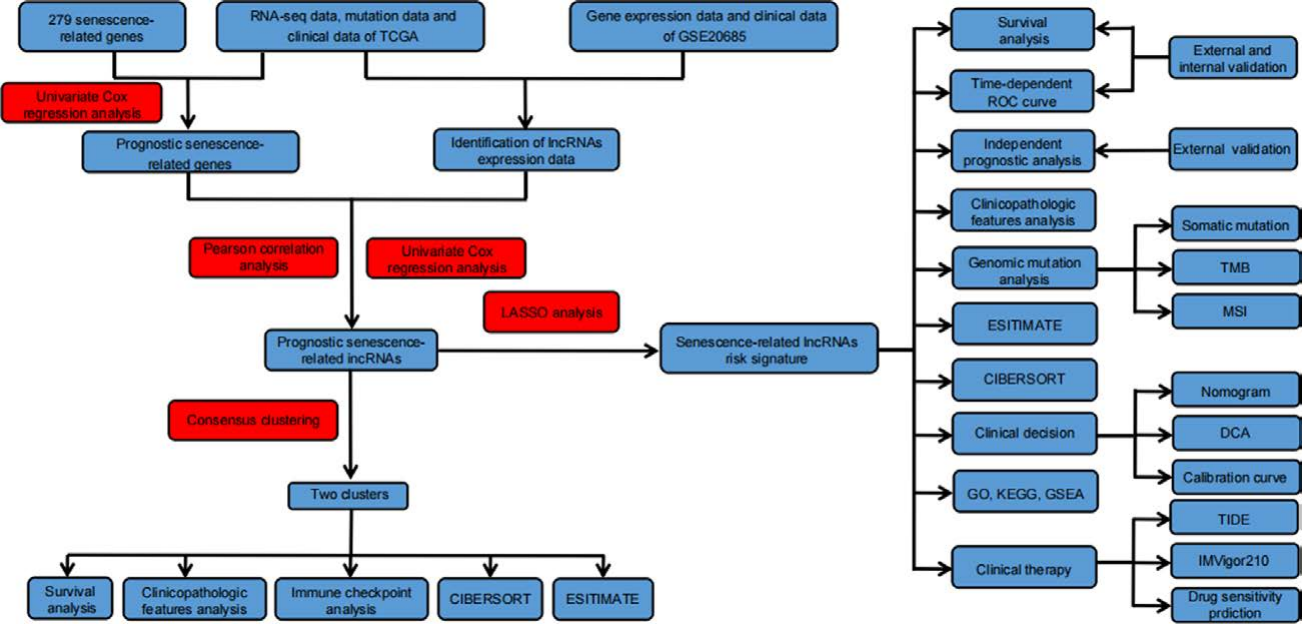
The flow chart of the study.

**Figure 2.**
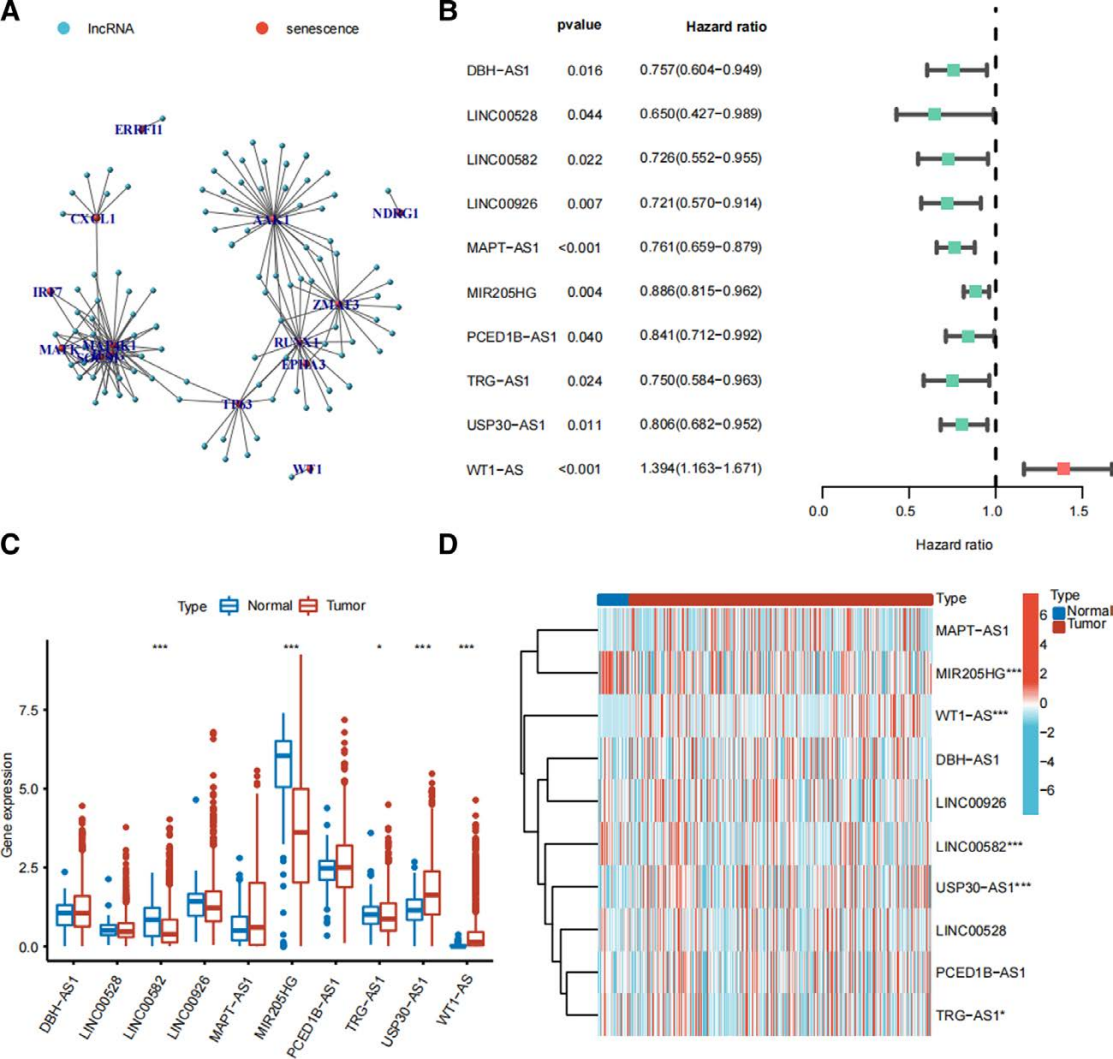
Screening of SRlncRNAs. (A) Co-expression network diagram of senescence-relevant genes and SRlncRNAs. (B) Forest plot shows ten prognostic SRlncRNAs identified by univariate Cox regression analysis. The boxplot (C) and heatmap (D) demonstrate the expression levels of the ten prognostic SRlncRNAs between the tumor and normal tissue. SRlncRNAs: Senescence-relevant long noncoding RNAs. *P < .05, **P < .01, and ***P < .001.

The research flowchart is presented in the Figure 1. Table 1 demonstrated clinicopathological information of BRCA patients from 2 databases. The prognostic senescence-relevant genes with the SRlncRNAs are displayed in the Figure 2A. The forest map shows the ten prognostic SRlncRNAs screening by univariate Cox regression analysis (Fig. 2B). Based on hazard ratio, WT1 − AS is regarded as deleterious, and the remaining SRlncRNAs are all protective. The expression levels of the 10 prognostic SRlncRNAs in the BRCA and normal tissue are showed in the Figure 2C and D.

**Table 1:** Clinicopathological features of breast cancer patients from TCGA and GSE20685.

Clinicopathological features	TCGA	%	GSE20685	%
Total	893	100	327	100
Age				
<65 yr	646	72.34	259	79.20
>=65 yr	247	27.66	68	20.80
Stage				
I	159	17.81	/	/
II	521	58.34	/	/
III	196	21.95	/	/
IV	17	1.90	/	/
T				
T1	235	26.32	101	30.89
T2	529	59.24	188	57.49
T3	98	10.97	26	7.95
T4	31	3.47	12	3.67
M				
M0	876	98.10	319	97.55
M1	17	1.90	8	2.45
N				
N0	438	49.05	137	41.90
N1	299	33.48	87	26.61
N2	103	11.53	63	19.27
N3	53	5.94	40	12.23
Follow-up time				
<5 yr	670	75.03	56	17.13
>=5 yr	223	24.97	271	82.87
Survival status				
Survival	769	86.11	244	74.62
Death	124	13.89	83	25.38

### 3.2. Comprehensive analysis of the 2 SRlncRNAs clusters

**Figure 3.**
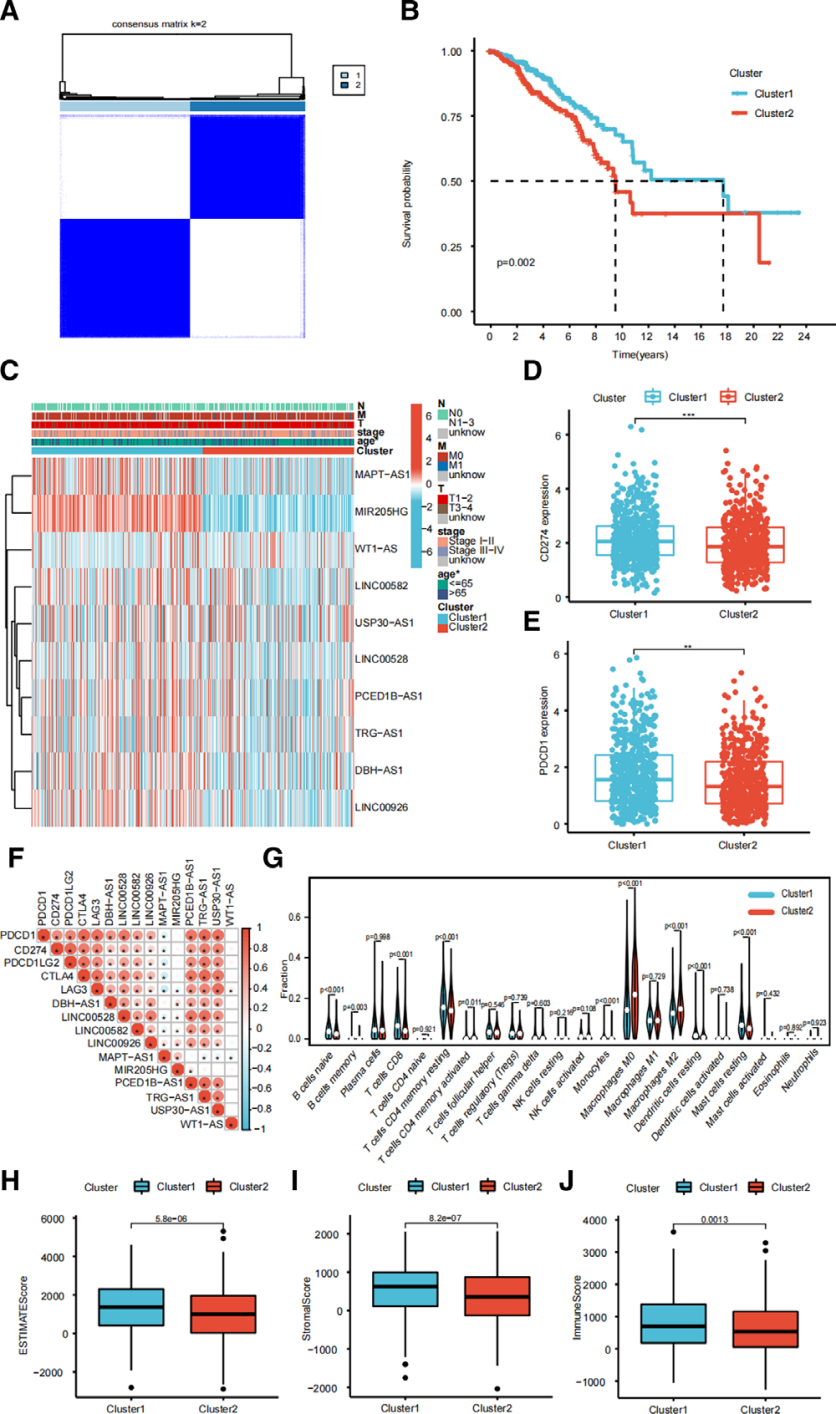
Consensus clustering of ten prognostic SRlncRNAs. (A) TCGA-BRCA cohort is classified into 2 clusters by consensus clustering. (B) BRCA patients of cluster 2 have significantly poorer OS. (C) Heatmap of clinicopathologic features analysis for the 2 clusters. The expression levels of CD274 (D) and PDCD1 (E) in the 2 clusters. (F) The relationship of the expression levels of ICIs and SRlncRNAs. (G) The immune infiltration levels of 22 immune cell types between the 2 clusters. The differences in ESTIMATEScore (H), StromalScore (I), and ImmuneScore (J) between the 2 clusters. BRCA = breast cancer, ICIs = immune checkpoint inhibitors, OS = overall survival, SRlncRNAs = senescence-relevant long noncoding RNAs, TCGA = The Cancer Genome Atlas. *P < .05, **P < .01, and ***P < .001.

The preferable clustering stability appears when we divide the TCGA cohort into 2 clusters (Fig. 3A). The cluster 2 has a worse OS (Fig. 3B; *P* = .002). Figure 3C shows the differences of clinicopathologic features between the 2 clusters.

We stay focused on the differences of immune status between the 2 clusters. BRCA patients of cluster 1 have higher expression level of CD274 (Fig. 3D; *P* < .001) and PDCD1 (Fig. 3E; *P* < .01). The correlation between the SRlncRNAs and immune checkpoints is presented in Figure 3F. Figure 3G shows the results of CIBERSORT that cluster 1 have higher infiltration levels of naive B cells, memory B cells, CD8 T cells, resting memory CD4 T cells, monocytes, resting dendritic cells, and resting mast cells, while cluster 2 have higher infiltration levels of activated memory CD4 T cells, M0 macrophages, and M2 macrophages. Eventually, cluster 1 have higher ESTIMATEScore (Fig. 3H), StromalScore (Fig. 3I), and ImmuneScore (Fig. 3J).

### 3.3. Establishment and validation of SRlncRNAs signature

**Figure 4.**
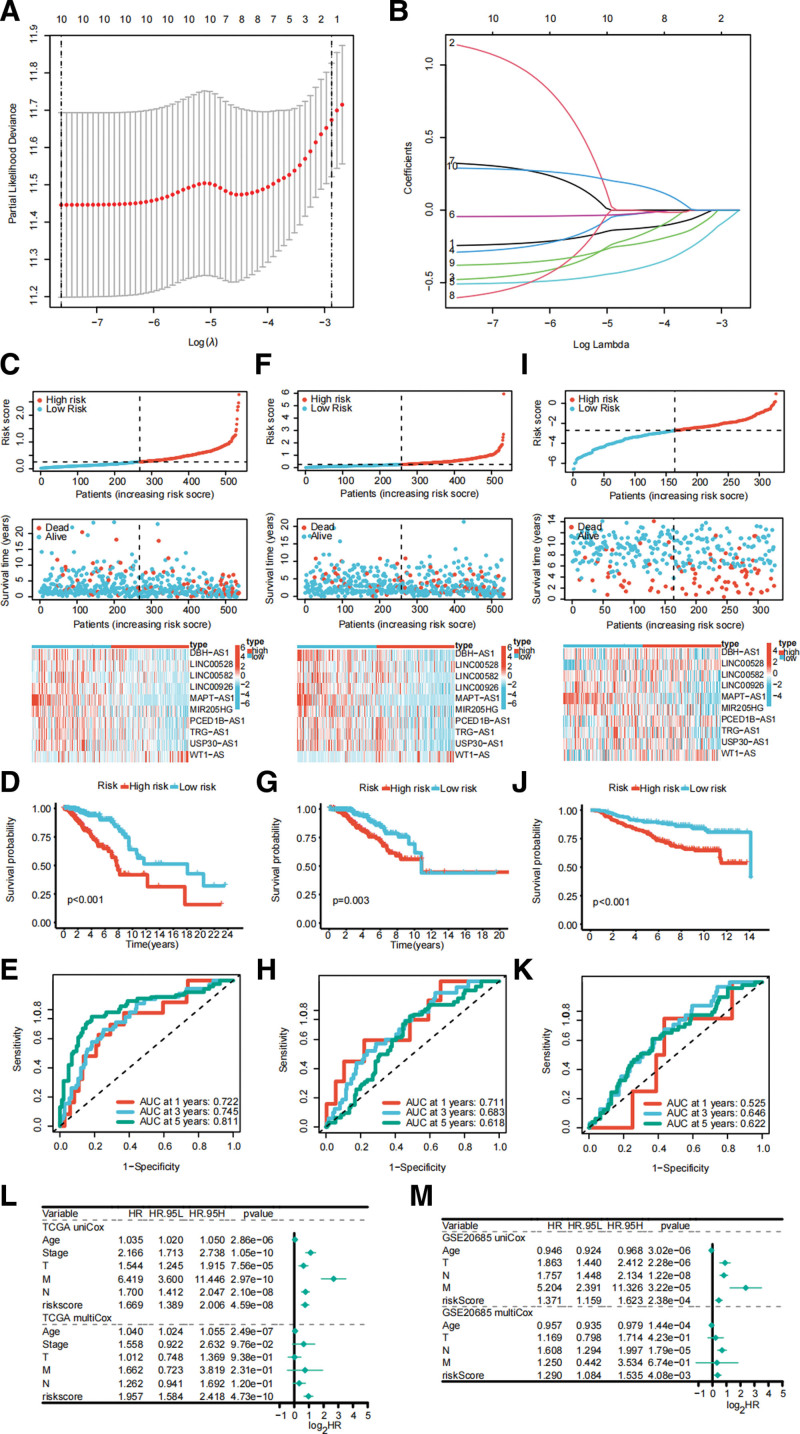
Development and validation of the SRlncRNAs signature. (A and B) LASSO analysis for the development of the SRlncRNAs signature. (C) The risk score, survival status, and expression levels of SRlncRNAs in TCGA training cohort. (D) Kaplan–Meier curves of TCGA training cohort. (E) Time-dependent ROC curves demonstrate the AUC value of TCGA training cohort. Validation the above results in TCGA internal validation cohort (F−H), and GSE20685 external validation cohort (I−K). Independent prognostic analysis of the risk score in TCGA cohort (L) and GSE20685 cohort (M). AUC = area under the curve, LASSO = least absolute shrinkage and selection operator, ROC = receiver operating characteristic, SRlncRNAs = senescence-relevant long noncoding RNAs, TCGA = The Cancer Genome Atlas.

After the examination of LASSO regression analysis, all of the 10 SRlncRNAs were included for the construction of the SRlncRNAs signature (Fig. 4A−B). The TCGA cohort was classified into training and or internal validation cohort. The risk score was acquired by the above-mentioned method, and BRCA patients were classified into low- or high-risk group based on median. The risk score ranking diagram, survival status, and heatmap of the expression levels of ten SRlncRNAs of TCGA training cohort are showed in Figure 4C. The Kaplan–Meier curves shows that high-risk BRCA have significantly worse OS (Fig. 4D; *P* < .001). Meanwhile, we confirm that our SRlncRNAs signature retains its prognosis prediction effectiveness under different clinical features (see Fig. S1, http://links.lww.com/MD/J311, Supplemental Content, which illustrates subgroup survival analysis). The 1-, 3-, and 5-year ROC curves also show that ideal AUC values (1-year: 0.722; 3-year: 0.745; 5-year: 0.811) in predicting BRCA outcomes (Fig. 4E). These results were obtained in the internal and external validation cohort (Fig. 4F−K). Subgroup analysis of external validation cohort also showed that high-risk BRCA had significantly worse OS under most clinical status except in patients of >65 years, M1, and T3-4, which was probably caused by the limited sample size in these subgroups (see Fig. S2, http://links.lww.com/MD/J312, Supplemental Content, which illustrates subgroup survival analysis). Eventually, Cox regression analyses verify that SRlncRNAs signature is a deleterious independent prognostic factor of BRCA in TCGA cohort (Fig. 4L) and GSE20685 cohort (Fig. 4M).

### 3.4. Transcriptome analysis revealing worse immune status in high-risk BRCA

**Figure 5.**
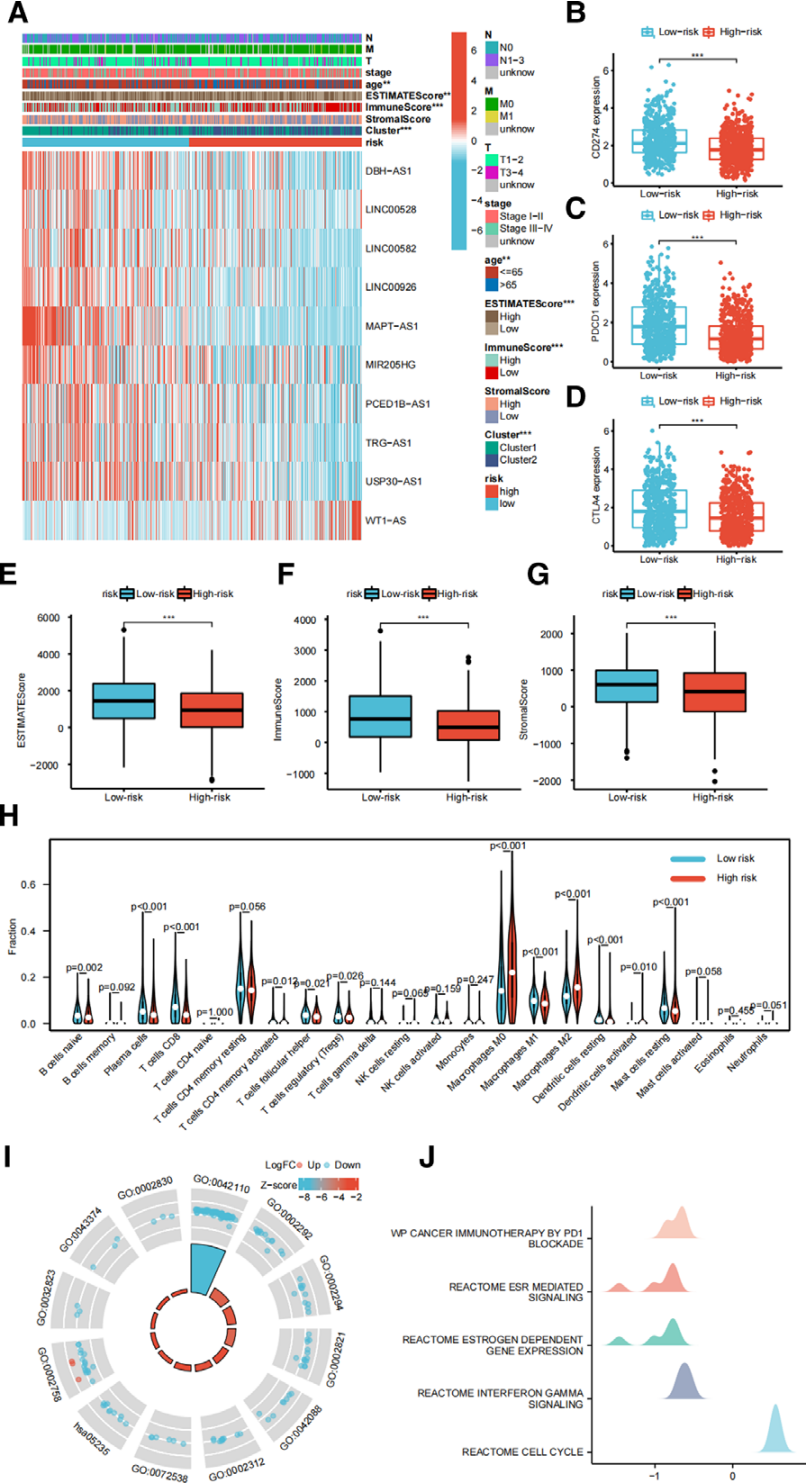
Transcriptome analysis reveals different immune status in the 2 risk groups. (A) Heatmap of clinicopathologic features analysis for the 2 risk groups. The expression levels of CD274 (B), PDCD1 (C), and CTLA4 (D) in the 2 risk groups. The differences in ESTIMATEScore (E), StromalScore (F), and ImmuneScore (G) between the 2 risk groups. (H) The immune infiltration levels of 22 immune cell types between the 2 risk groups. (I) The circle map of GO and KEGG analysis. (J) The ridge plot of GSEA. GO = gene ontology, GSEA = gene set enrichment analysis, KEGG = Kyoto Encyclopedia of Genes and Genomes. *P < .05, **P < .01, and ***P < .001. GO:0042110, T cell activation; GO:0002292, T cell differentiation involved in immune response; GO:0002294, CD4-positive, alpha-beta T cell differentiation involved in immune response; GO:0002821, positive regulation of adaptive immune response; GO:0042088, T-helper 1 type immune response; GO:0002312, B cell activation involved in immune response; GO:0072538, T-helper 17 type immune response; GO:0002758, innate immune response-activating signal transduction; GO:0032823, regulation of natural killer cell differentiation; GO:0043374, CD8-positive, alpha-beta T cell differentiation; GO:0002830, positive regulation of type 2 immune response; hsa05235, PD-L1 expression and PD-1 checkpoint pathway in cancer.

The heatmap shows the differences of clinicopathologic characteristics of the 2 risk groups (Fig. 5A). In turn, we investigated the differences of risk score between patients from different clinical groups, and found that patients of cluster 2, age >65 years, low ESTIMATEScore, low ImmuneScore, low StromalScore, and stage III to IV have higher risk score, while no differences of risk score were found between different T, M, N stages (see Fig. S3, http://links.lww.com/MD/J313, Supplemental Content, which illustrates subgroup analysis of the risk score). Further, we compared the expression levels of the immune checkpoints and found that low-risk BRCA patients have higher CD274 (Fig. 5B), PDCD1 (Fig. 5C), and CTLA4 (Fig. 5D) expression level. In the meantime, low-risk BRCA patients have higher ESTIMATEScore (Fig. 5E), ImmuneScore (Fig. 5F), and StromalScore (Fig. 5G). The high-risk BRCA patients have higher infiltration levels of M0 macrophages, M2 macrophages, and activated dendritic cells, while low-risk BRCA patients have higher infiltration levels of naive B cells, plasma cells, CD8 T cells, activated memory CD4 T cells, T follicular helper cells, Tregs, M1 macrophages, resting dendritic cells, activated dendritic cells, and resting mast cells (Fig. 5H). Further, we explored the relationship between the risk score and the immune cell infiltration levels. It was showed that risk score is positively correlated with the infiltration levels of M0 macrophages, M2 macrophages, activated mast cells, resting NK cells, and activated dendritic cells, and negatively correlated with the infiltration levels of M1 macrophages, resting mast cells, naive B cells, resting dendritic cells, resting memory CD4 T cells, CD8 T cells, T follicular helper cells, gamma delta T cells, Tregs, plasma cells, and activated memory CD4 T cells (see Fig. S4, http://links.lww.com/MD/J314, Supplemental Content, which illustrates correlation analysis of the risk score and immune cells infiltration). GO and KEGG analysis shows that DEGs of the 2 risk groups are mainly enriched in negative regulation of “T cell activation,” “T cell differentiation involved in immune response,” “CD4-positive, alpha-beta T cell differentiation involved in immune response,” “positive regulation of adaptive immune response,” “T-helper 1 type immune response,” “B cell activation involved in immune response,” “T-helper 17 type immune response,” “innate immune response-activating signal transduction,” “regulation of natural killer cell differentiation,” “CD8-positive, alpha-beta T cell differentiation,” “positive regulation of type 2 immune response,” and “PD-L1 expression and PD-1 checkpoint pathway in cancer” (Fig. 5I). GSEA demonstrates that low-risk BRCA patients are related to “Cancer immunotherapy by PD1 blockade,” “ESR mediated signaling,” “Estrogen dependent gene expression,” and “Interferon gamma signaling,” while high-risk BRCA patients are related to “Cell cycle” (Fig. 5J).

### 3.5. Genome analysis revealing lower mutation frequency and higher TMB in high-risk BRCA patients

**Figure 6.**
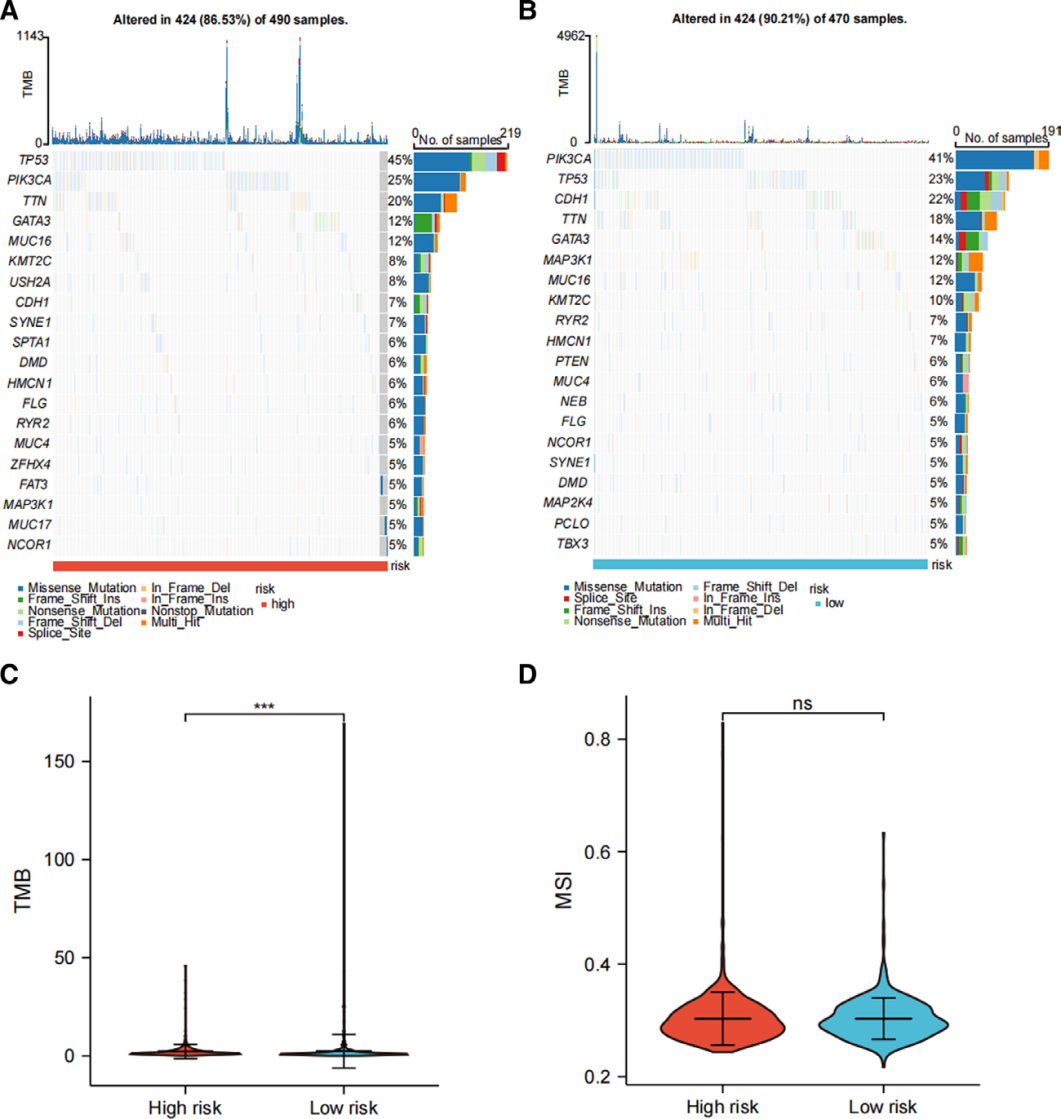
Genome analysis reveals the difference between the 2 risk groups. The oncoplot show top 20 genes in the high-risk (A) and low-risk (B) groups in terms of mutation frequency. The different TMB (C) and MSI (D) scores between the 2 risk groups. MSI = microsatellite instability, TMB = tumor mutation burden. *P < .05, **P < .01, and ***P < .001.

The oncoplots show that high-risk BRCA have lower mutation frequency (Fig. 6A−B). The top 5 mutation genes of the high-risk group are TP53 (45%), PIK3CA (25%), TTN (20%), GATA3 (12%), and MUC16 (12%), and the top 5 mutation genes of the low-risk group are PIK3CA (41%), TP53 (23%), CDH1 (22%), TTN (18%), and GATA3 (14%) (Fig. 6A−B). The TMB of the high-risk group is significantly higher (Fig. 6C; *P* < .001), while no significant difference of MSI was found between the 2 risk groups (Fig. 6D).

### 3.6. Nomogram, calibration curves and DCA precisely predicting the prognosis of BRCA

**Figure 7.**
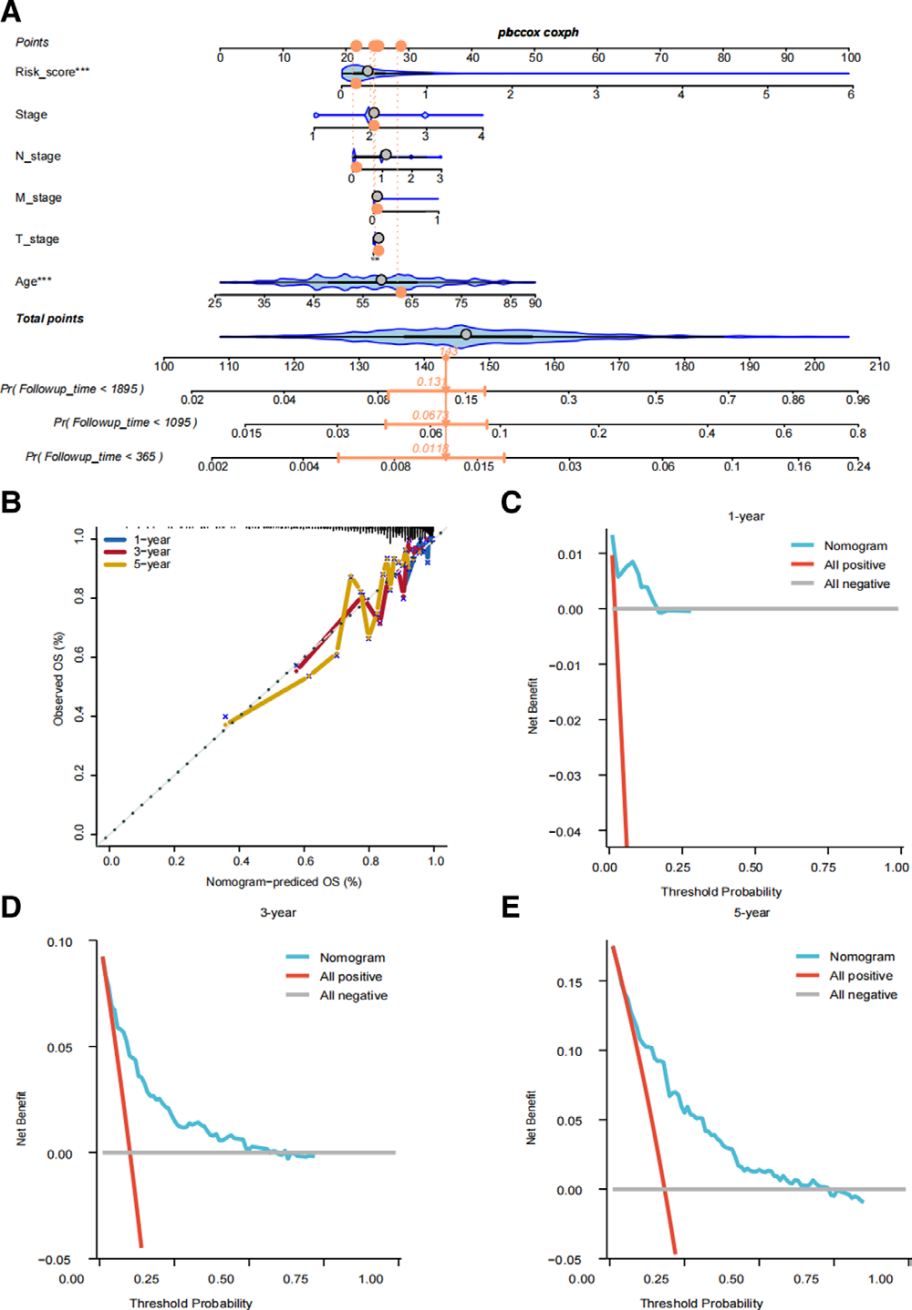
Construction of a nomogram combining SRlncRNAs signature and other clinicopathological factors for clinical decision. (A) Nomogram of SRlncRNAs signature and other clinicopathological factors. (B) One-, 3-, and 5-yr calibration curves estimating the precision of the nomogram. The 1- (C), 3- (D), 5- (E) yr DCA curves showing the clinical benefits. DCA = decision curve analysis, SRlncRNAs = senescence-relevant long noncoding RNAs.

We combined all the clinicopathologic characteristics and developed a nomogram for better clinical use (Fig. 7A). The calibration curves demonstrate the ideal ability of the nomogram in predicting the prognosis of BRCA patients (Fig. 7B). The 1-, 3-, 5-DCA curves also demonstrate that the nomogram performs well in predicting the outcome of BRCA (Fig. 7C−E).

### 3.7. High-risk patients having higher response rate to ICIs

**Figure 8.**
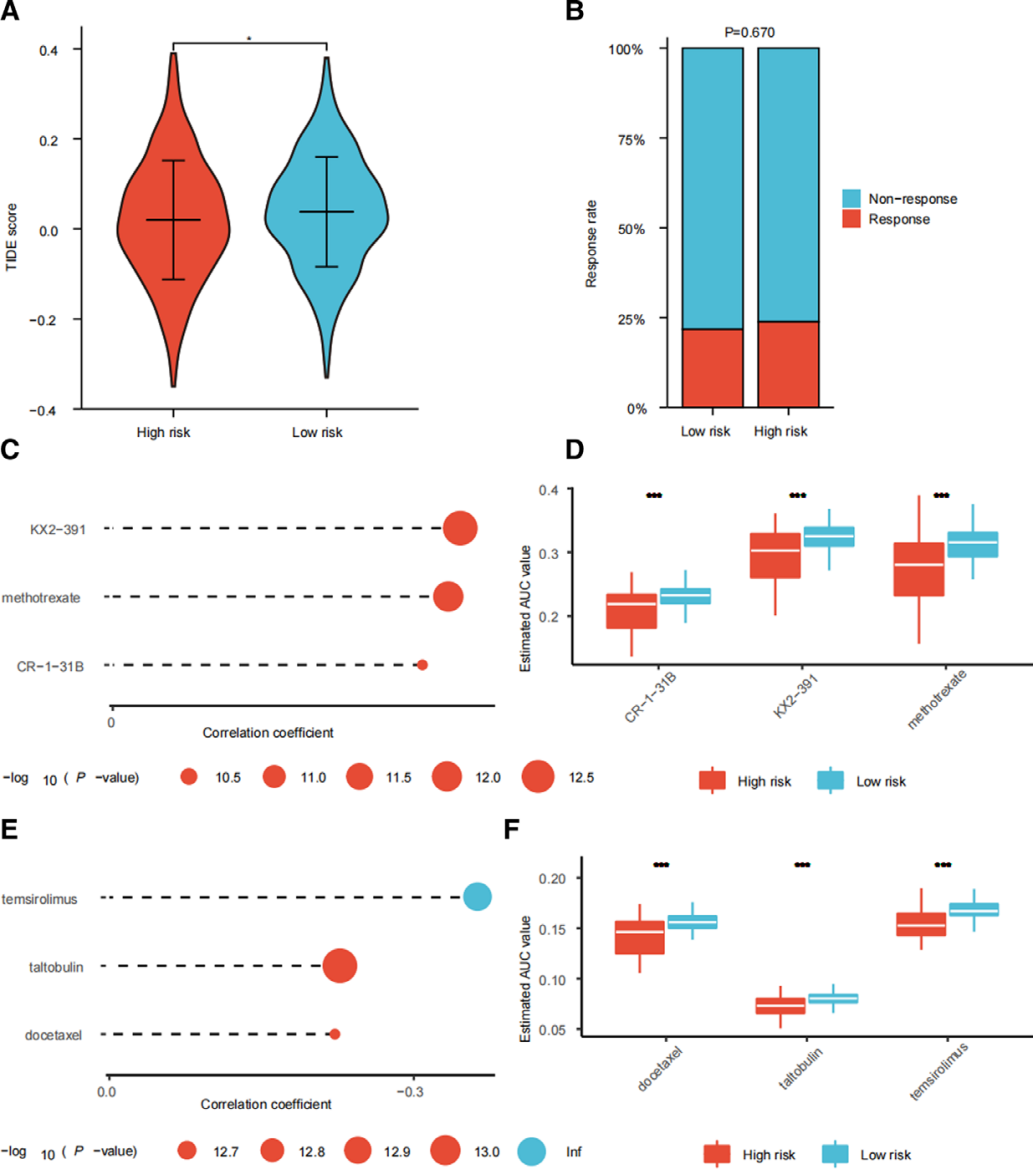
The response to immunotherapy and drug susceptibility analysis of the 2 risk groups. (A) The TIDE score of the 2 risk groups. (B) Predicting the response to immunotherapy using IMvigor-210 cohort. Drug susceptibility analysis predicting sensitive compounds to BRCA using CTRP (C) and PRISM (D) databases. BRCA = breast cancer; CTRP = Cancer Therapeutics Response Portal resource, PRISM = Profiling Relative Inhibition Simultaneously in Mixtures, TIDE = tumor immune dysfunction and exclusion. *P < .05, **P < .01, and ***P < .001.

TIDE score of each BRCA patient were obtained and comparison was made between the 2 risk groups. We find that high-risk patients have lower TIDE score (Fig. 8A), which means high-risk patients are more susceptive to ICIs. We extended the SRlncRNAs signature to IMvigor-210 cohort. Consistent with the previous result, high-risk patients have higher response rate to ICIs, though this result is not statistically significant (Fig. 8B). Drug susceptibility analysis yielded 3 CTRP-derived compounds (KX2-391, methotrexate, and CR-1-31B) (Fig. 8C−D) and 3 PRISM-derived compounds (temsirolimus, taltobulin, and docetaxel) (Fig. 8E−F). For the 6 compounds, high-risk BRCA patients have lower AUC values, which means they are more sensitive to these compounds.

## 4. Discussion

Currently, approximately 2.3 million patients worldwide are newly diagnosed with BRCA each year, which has become a huge burden of human health.^[1]^ The rapid development of immunotherapy, especially ICIs, has brought a new light to the prognosis of BRCA patients. However, ICIs have low response rates and high toxicity to some BRCA patients.^[24,25]^ How to screen the population for potential benefit of ICIs is a challenge that deserves the attention of researchers. The advent of high-throughput sequencing has allowed the mining of a mass of cancer-relevant biomarkers, offering the possibility to address the above-mentioned challenge. Cellular senescence is a crucial phenotype of disease, the accumulation and mutation of senescent cells allow the occurrence of immune escape, which is one of the crucial mechanisms in the development of malignant tumors.^[26,27]^ However, traditional studies of cellular senescence are mainly focused on the genetic level, the role of SRlncRNAs in the prognosis and efficacy of immunotherapy of BRCA remains to be elucidate. Here, we comprehensively explored the crucial role of SRlncRNAs signature in BRCA patients at the transcriptomic and genomic levels.

After the screening of the univariate Cox regression analysis, 10 SRlncRNAs were identified. Among them, LINC00582, MIR205HG, and TRG − AS1 were significantly upregulated in the normal tissue, while USP30 − AS1 and WT1 − AS were significantly upregulated in BRCA. Chen et al found that DBH − AS1 promotes the development of melanoma via miR-233-3p/IGF-1R/Akt axis.^[28]^ Chu et al reported that LINC00926 suppresses BRCA proliferation via inhibition of PGK1-mediated Warburg effect.^[29]^ Wang et al found that BRCA patients with high expression level of MAPT-AS1 are associated with better OS.^[30]^ Xu et al reported that MIR205HG could predict the efficacy of neoadjuvant chemotherapy for advanced BRCA.^[31]^ Liu et al found CED1B-AS1 promotes the growth of colorectal adenocarcinoma via the miR-633/HOXA9 axis.^[32]^ Sun et al found that TRG-AS1 stimulates liver cancer proliferation by sponging miR-4500 to modulate BACH1.^[33]^ Li et al reported USP30-AS1 sponges miR-765 and regulates the proliferation of colon cancer.^[34]^ Wang et al found that WT1-AS inhibits triple-negative BRCA cell invasion by downregulating the transforming TGF-β.^[35]^ However, there are no studies reporting the effect of the remaining SRlncRNAs in cancer. By consensus clustering, TCGA-BRCA cohort was classified into 2 clusters. Cluster 1 had better OS and higher expression levels of CD274 and PDCD1. The expression levels of SRlncRNAs were correlated with the expression levels of immune checkpoints, suggesting a potential association with the efficacy of ICIs. Cluster 1 had an ideal immune microenvironment with higher infiltration levels of immunologic effector cells like CD8 T cells, and monocytes, while cluster 2 had an immunosuppressive microenvironment with higher infiltration levels of immunosuppressive cells like M2 macrophages. The results of ESITIMATE algorithm also further verified the above conclusion.

After LASSO regression analysis, all of the ten SRlncRNAs were used for the establishment of the SRlncRNAs signature and BRCA patients were classified into 2 risk groups. High-risk patients had worse OS in training cohort, which was also verified by the internal and external validation cohorts. The SRlncRNAs signature had ideal performance in predicting BRCA prognosis as shown by ROC curves. We also explored the immune status of the 2 risk groups, and found the higher expression levels of immune checkpoints, ESITIMATE algorithm scores and infiltration levels of immunologic effector cells. Better immune status explains the better OS of the low-risk BRCA. GO, KEGG and GSEA demonstrated that SRlncRNAs signature is related to some tumorigenesis-relevant and immune-response related pathways. The above results reveal the differences in prognosis and immune status of the 2 risk groups by transcriptome analysis, suggesting that the SRlncRNAs signature may correlate with the response to immunotherapy in BRCA.

Next, we tried to decode the differences between the risk 2 groups at the genomic level. A higher mutation frequency of TP53 was found in the high-risk BRCA patients. TP53 is a transcription factor that regulates the cellular responses that together prevent tumorigenesis, such as apoptotic cell death and DNA damage repair.^[36]^ TP53 mutation not only promotes the tumorigenesis but also enhances resistance of DNA damage drugs, which contributes the poor OS of patients.^[37]^ Conversely, we found a higher mutation frequency of PIK3CA in the low-risk group. PIK3CA mutation is recognized as a crucial oncogenic drivers in BRCA.^[38]^ The result of NCI-MATCH ECOG-ACRIN Trial (EAY131) Subprotocol Z1F showed that copanlisib, a PI3K inhibitor, achieves an objective response rate of 16% in HER2 (+) BRCA.^[39]^ Genome analysis reveals the different gene mutation status between the 2 risk groups and explains the difference in prognosis of BRCA patients to some extent.

For better clinical decision, a nomogram was developed by combining the SRlncRNAs signature with other clinicopathologic features. This nomogram allows clinicians to accurately predict the prognosis and clinical benefit of BRCA patients at 1-, 3-, and 5-year intervals.

The different immune status of the 2 risk groups reveals that they may have different response rates to immunotherapy. Thus, we calculated the TIDE score of the 2 groups, and found that high-risk BRCA patients have significantly lower TIDE score, which means that they are more sensitive to ICIs. Recently, significant progress has been achieved in screening small molecule compounds for the treatment of BRCA with the advent of drug databases.^[40,41]^ We obtained 6 compounds that may be more sensitive to high-risk BRCA patients by screening of the CTRP and PRISM databases. KX2 − 391 is an inhibitor of Src and pretubulin, which could inhibit BRCA growth and metastasis.^[42]^ Methotrexate has broad-spectrum antitumor activity. Lu et al found that methotrexate combined with cyclophosphamide could achieve a clinical benefit rate of 31.2% at 24 weeks for advanced BRCA.^[43]^ Temsirolimus is an mTOR inhibitor. The combination treatment of AEE788 and temsirolimus could inhibit phosphorylation of mTOR, thereby restraining the proliferation of BRCA cells.^[44]^ Docetaxel could disrupt the microtubule meshwork, thereby destroying the cancer cells. Sheng et al found that docetaxel-induced Ccl3 could promote the proinflammatory macrophage polarization and subsequently accelerate phagocytosis of tumor cells.^[45]^

In recent years, lncRNA-based prognostic signature for BRCA have been emerging. Lv et al developed an m6A-related lncRNA signature as predictive biomarkers for BRCA.^[46]^ However, the AUCs of this signature in training cohort were remained to be improved (1-year AUC: 0.677, 3-year AUC: 0.678, 5-year AUC: 0.692). Shen et al identified an immune-related lncRNA prognostic signature for BRCA and found the high-risk group exhibited worse OS than those in the low-risk group.^[47]^ However, this study was only based on the TCGA database and lacked external validation. The SRlncRNA signature not only had relatively higher AUCs (1-year AUC: 0.722, 3-year AUC: 0.745, 5-year AUC: 0.811) but also were verified by external validation cohort. Currently, Xing et al constructed a senescence-related signature for predicting the prognosis of BRCA,^[48]^ however, this work was based on the gene expression data and can not explain the role of noncoding RNAs in BRCA.

The main innovation of this work was that it comprehensively explored and confirmed the relationship between SRlncRNAs and the prognosis and immunotherapy of BRCA based on multi-omics data, which demonstrated a favorable clinical application potential.

The SRlncRNAs signature also has some limitations to state. First, due to the lack of tumor specimens, it is difficult for us to verify these conclusions through biological experiments. Second, though we have validated the SRlncRNAs signature by internal validation and external validation cohorts, multicenter and multiracial cohorts are still needed to verify and refine the signature before clinical applications.

## 5. Conclusions

We developed and verified an original SRlncRNAs signature by multi-omics analysis, which could serve as a prognosis and immunotherapy predictor for BRCA patients.

## Acknowledgments

We thank for Bioinfo composer and FigureYa for their helpful suggestions.

## Author contributions

**Conceptualization:** Jie Ji.

**Data curation:** Yu Ziyi.

**Formal analysis:** Yu Ziyi.

**Funding acquisition:** Jie Ji.

**Project administration:** Jie Ji.

**Validation:** Yu Ziyi, Yanhui Zhu.

**Visualization:** Yu Ziyi.

**Writing – original draft:** Yu Ziyi, Jie Ji.

**Writing – review & editing:** Yanhui Zhu, Jie Ji.

## Supplementary Material










