# Supplementary material for: Multi-omics analysis revealing a senescence-relevant lncRNAs signature for the assessment of response to immunotherapy for breast cancer
Source: Medicine (Baltimore). 2023 Jul 14;102(28):e34287. doi: 10.1097/MD.0000000000034287 (PMC10344520; doi:10.1097/MD.0000000000034287)
Supplement: Supplementary file 1 [file medi-102-e34287-s001.pdf]

Table S1. The list of senescence-relevant genes.
ACLY
AAK1
ABI3
ADCK5
AKR1B1
AGT
AKT1
ALOX15B
AR
ARPC1B
ASF1A
ASPH
ATF7IP
ATM
AURKA
AXL
BAG3
BHLHE40
BCL6
BLK
BLVRA
BMI1
BRAF
BRD7
BRCA1
BTG3
C11orf31
CAV1
CBX7
CBX8
CCND1
CDK1
CDK18
CDK2AP1
CDK6
CDK4
CDKN1A
CDKN1C
CDKN1B
CDKN2A
CDKN2AIP
CDKN2B
CENPA

CEBPB
CHEK1
CKB
CPEB1
CSNK1A1
CTNNAL1
CSNK2A1
CXCL1
DDB2
CYR61
DEK
DGCR8
DHCR24
DLX2
DHX9
DPY30
DUSP3
DUSP16
E2F1
EHF
ENDOG
EPHA3
ERRFI1
ETS1
ETS2
EWSR1
FASTK
EZH2
FBXO31
FOXM1
FOS
FOXO3
FXR1
G6PD
GAPDH
GKN1
GATA4
GNG11
GLB1
GRK6
HDAC4
HDAC1
HEPACAM
HJURP

HIVEP1
HK3
HMGB1
HRAS
HSPA5
HSPB2
ID1
ID4
IGFBP1
IFNG
IGFBP3
IGFBP6
IGFBP5
IL1A
IL8
ING1
ING2
IRF3
IRF5
IRF7
ITPK1
ITGB4
ITPKB
ITSN2
KCNJ12
KDM4A
KDM5B
KIAA1524
KL
KSR2
LATS1
LEO1
LGALS3
LIMA1
LIMK1
MAGEA2
MAGOH
MAD2L1
MAGOHB
MAP2K1
MAP2K3
MAP2K2
MAP2K6
MAP3K6

MAP2K7
MAP4K1
MAP3K7
MAPK12
MAPKAPK5
5-Mar
MAPK14
MAST1
MATK
MCL1
MDH1
MCRS1
MECP2
MOB3A
MMP9
MORC3
MORF4
MXD4
MVK
MYC
MYLK
NADK
NANOG
NDRG1
NEK1
NEK4
NEK6
NFE2L2
NINJ1
NOTCH3
NOX4
NR2E1
NTN4
NUAK1
OTX2
P3H1
PATZ1
PAK4
PBRM1
PCGF2
PDCD10
PDIK1L
PDZD2
PDPK1

PEBP1
PEX19
PIAS4
PIK3R5
PIK3C2A
PIM1
PLA2R1
PKM
PML
PNPT1
PMVK
POT1
POU5F1
PPM1B
PPM1D
PRMT6
PRKCH
PRKCD
PROX1
PRPF19
PSMB5
PTRF
PTTG1
PSMD14
RAD21
RAF1
RB1
RBP2
RBX1
RNASEL
RPS6KA6
RSL1D1
RUNX1
RUVBL2
SENP1
SENP2
SENP7
SERPINE1
SFN
SIK1
SGK1
SIN3B
SIRT1
SIRT6

SIX1
SLC13A3
SLC16A7
SMARCA4
SMG1
SMARCB1
SMURF2
SNAI1
SOCS1
SOD1
SORBS2
SOX2
SPIN1
SOX5
SP1
SPOP
SRC
SREBF1
SRSF1
STAT5B
STK32C
STK40
SUPT5H
SYK
TACC3
TERC
TBX2
TERF2
TERT
TFAP4
TFDP1
TGFB1I1
TLR3
TMSB4X
TNFSF13
TNFSF15
TOP1
TP63
TPR
TP53
TRIM28
TRPM8
TXN
TXNIP

UBTD1
TYK2
VENTX
USP1
VEGFA
WNT16
WNT2
WRN
WT1
XAF1
WWP1
YAP1
YPEL3
ZFP36
ZMAT3
ZNF148
