# Supplementary material for: Multi-omics analysis revealing a senescence-relevant lncRNAs signature for the assessment of response to immunotherapy for breast cancer
Source: Medicine (Baltimore). 2023 Jul 14;102(28):e34287. doi: 10.1097/MD.0000000000034287 (PMC10344520; doi:10.1097/MD.0000000000034287)
Supplement: Supplementary file 2 [file medi-102-e34287-s002.pdf]

Figure S1. Subgroup survival analysis of TCGA cohort. (A) Age>65. (B) Age≤65. (C) M0. (D) M1. (E) N0. (F) N1-3. (G) T1-2. (H) T3–4. (I) Stage I–II. (J) Stage III–IV. TCGA: The Cancer Genome Atlas

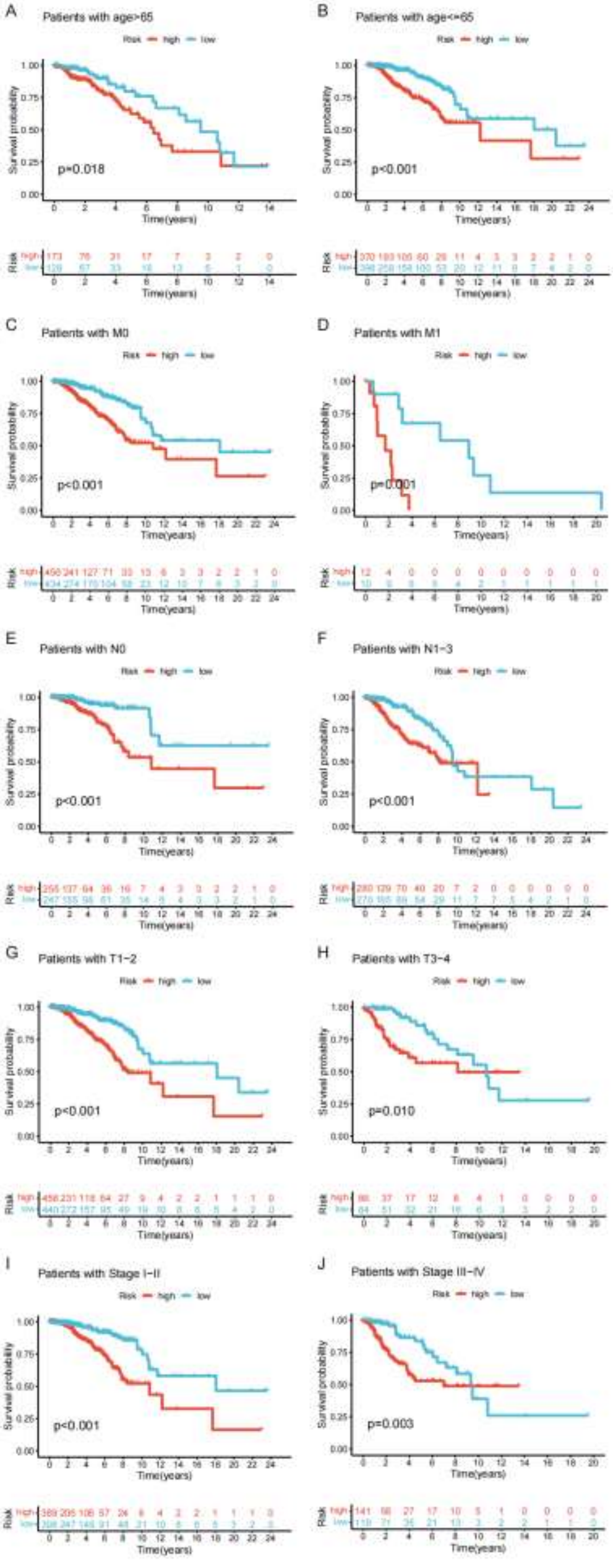
A
Patients with age>65
Risk — high — low
Survival probability
p=0.018
Time(years)
B
Patients with age<=65
p<0.001
C
Patients with M0
p<0.001
D
Patients with M1
p=0.001
E
Patients with N0
p<0.001
F
Patients with N1−3
p<0.001
G
Patients with T1−2
p<0.001
H
Patients with T3−4
p=0.010
I
Patients with Stage I−II
p<0.001
J
Patients with Stage III−IV
p=0.003
