# Supplementary figures and images for: Multi-omics analysis revealing a senescence-relevant lncRNAs signature for the assessment of response to immunotherapy for breast cancer
Source: Medicine (Baltimore). 2023 Jul 14;102(28):e34287. doi: 10.1097/MD.0000000000034287 (PMC10344520; doi:10.1097/MD.0000000000034287)

Figure S2. Subgroup survival analysis of GSE20685. (A) Age>65. (B) Age≤65. (C) M0. (D) M1. (E) N0. (F) N1-3. (G) T1-2. (H) T3-4.

A

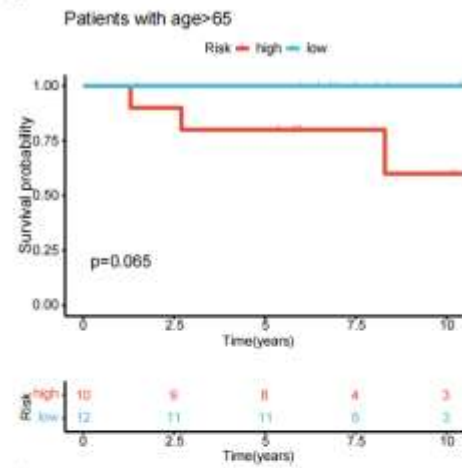

B

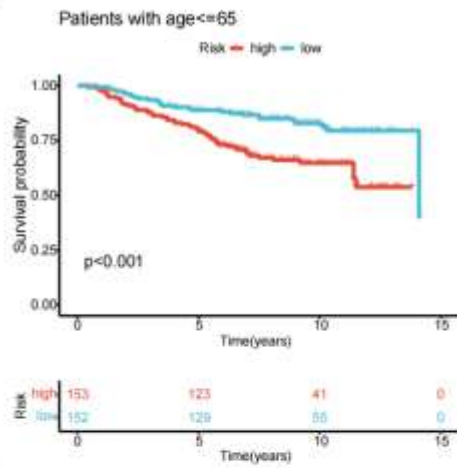

C

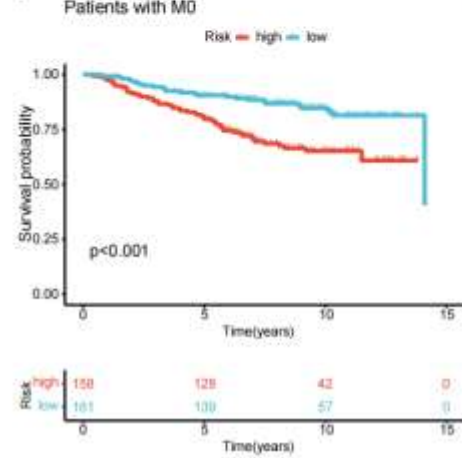

D

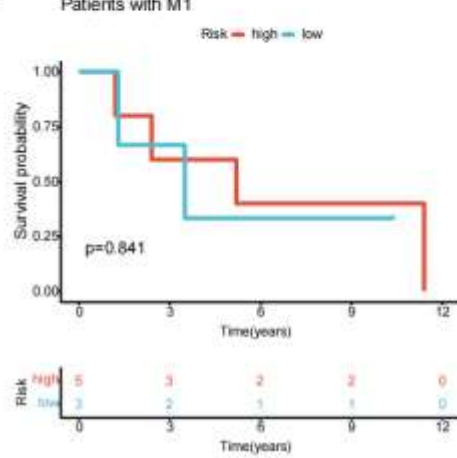

E

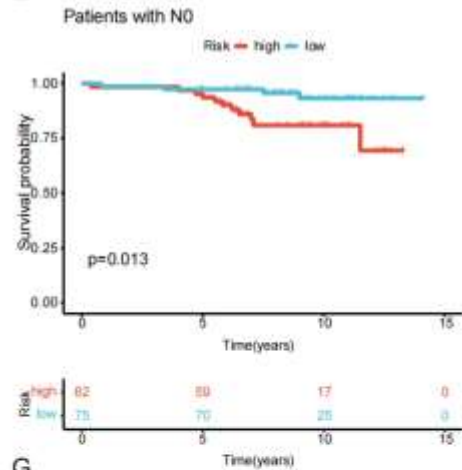

F

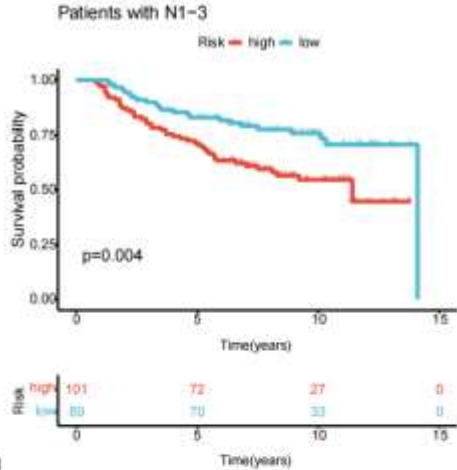

G

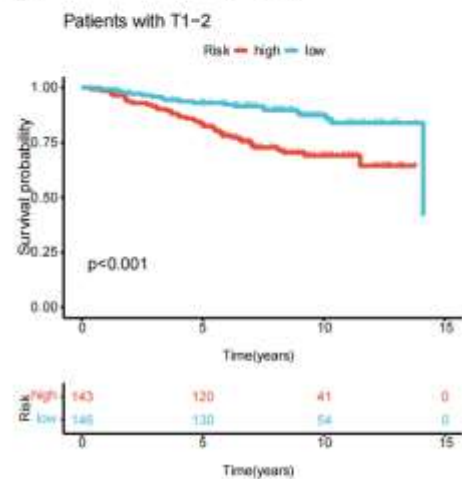

H

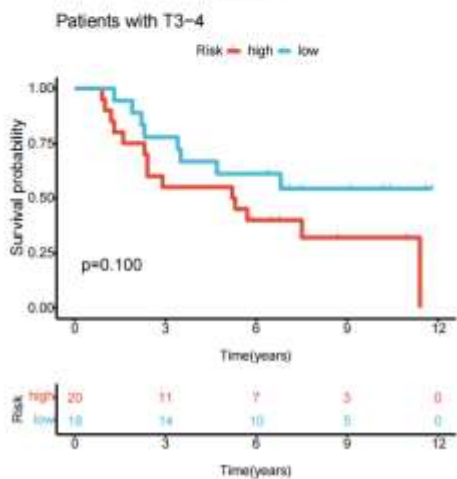

Supplement: Supplementary file 3 [file medi-102-e34287-s003.pdf]
