# Supplementary material for: Multi-omics analysis revealing a senescence-relevant lncRNAs signature for the assessment of response to immunotherapy for breast cancer
Source: Medicine (Baltimore). 2023 Jul 14;102(28):e34287. doi: 10.1097/MD.0000000000034287 (PMC10344520; doi:10.1097/MD.0000000000034287)
Supplement: Supplementary file 4 [file medi-102-e34287-s004.pdf]

Figure S3. Subgroup analysis of the risk score. (A) Cluster. (B) Age. (C) ESTIMATEScore. (D) ImmuneScore. (E) StromalScore. (F) Stage. (G) T. (H) M. (I) N.

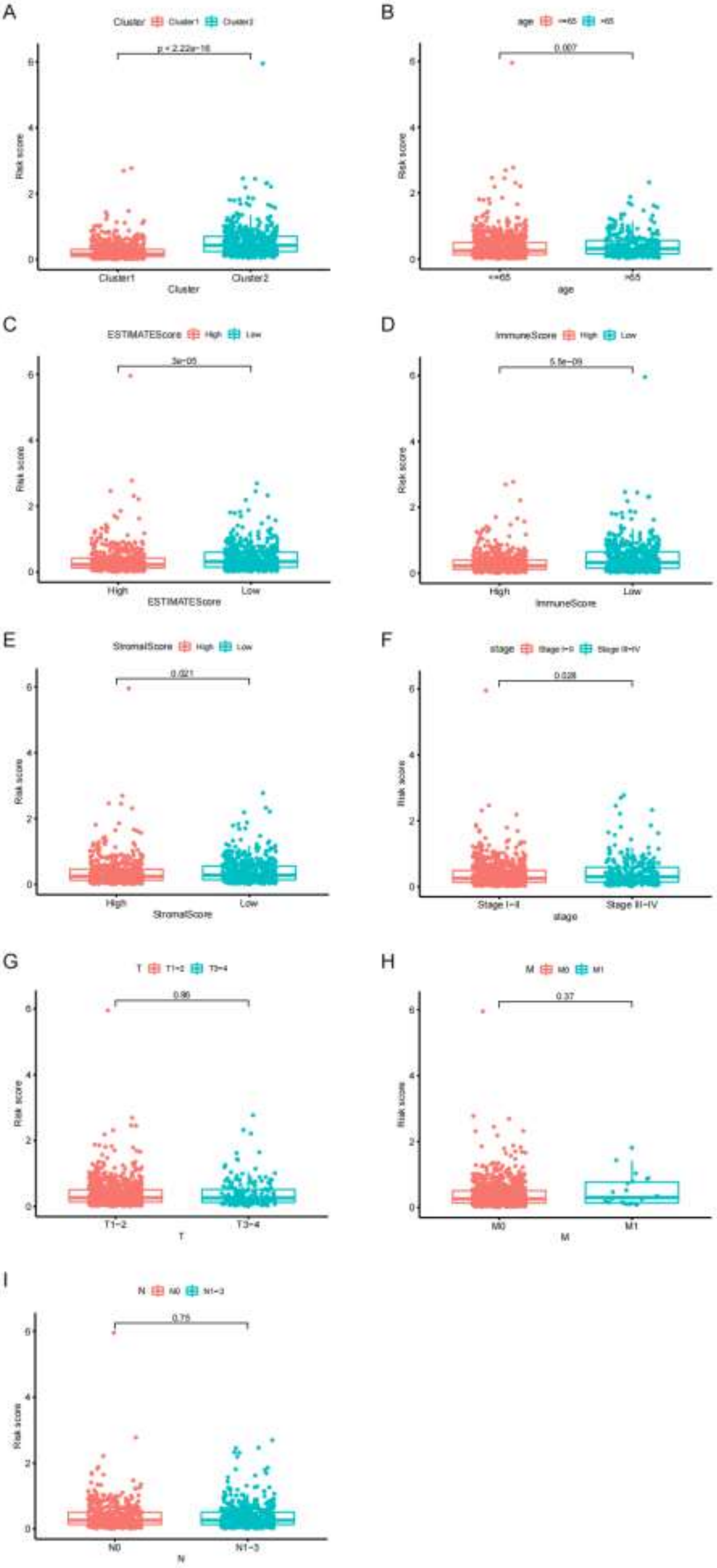
A
Cluster Cluster1 Cluster2
p < 2.22e−16
Risk score
Cluster1 Cluster2
Cluster
B
age <=65 >65
0.007
Risk score
<=65 >65
age
C
ESTIMATEScore High Low
3e−05
Risk score
High Low
ESTIMATEScore
D
ImmuneScore High Low
Risk score
High Low
ImmuneScore
E
StromalScore High Low
0.021
Risk score
High Low
StromalScore
F
stage Stage I−II Stage III−IV
0.028
Risk score
Stage I−II Stage III−IV
stage
G
T T1−2 T3−4
Risk score
T1−2 T3−4
T
H
M M0 M1
0.37
Risk score
M0 M1
M
I
N N0 N1−3
0.75
Risk score
N0 N1−3
N
