# Supplementary material for: Multi-omics analysis revealing a senescence-relevant lncRNAs signature for the assessment of response to immunotherapy for breast cancer
Source: Medicine (Baltimore). 2023 Jul 14;102(28):e34287. doi: 10.1097/MD.0000000000034287 (PMC10344520; doi:10.1097/MD.0000000000034287)
Supplement: Supplementary file 5 [file medi-102-e34287-s005.pdf]

Figure S4. Correlation analysis of the risk score and immune cells infiltration. (A) Macrophages M0. (B) Macrophages M1. (C) Macrophages M2. (D) Mast cells activated. (E) Mast cells resting. (F) NK cells resting. (G) B cells naive. (H) Dendritic cells activated. (I) Dendritic cells resting. (J) T cells CD4 memory resting. (K) T cells CD8. (L) T cells follicular helper. (M) T cells gamma delta. (N) T cells regulatory. (O) Plasma cells. (P) T cells CD4 memory activated.

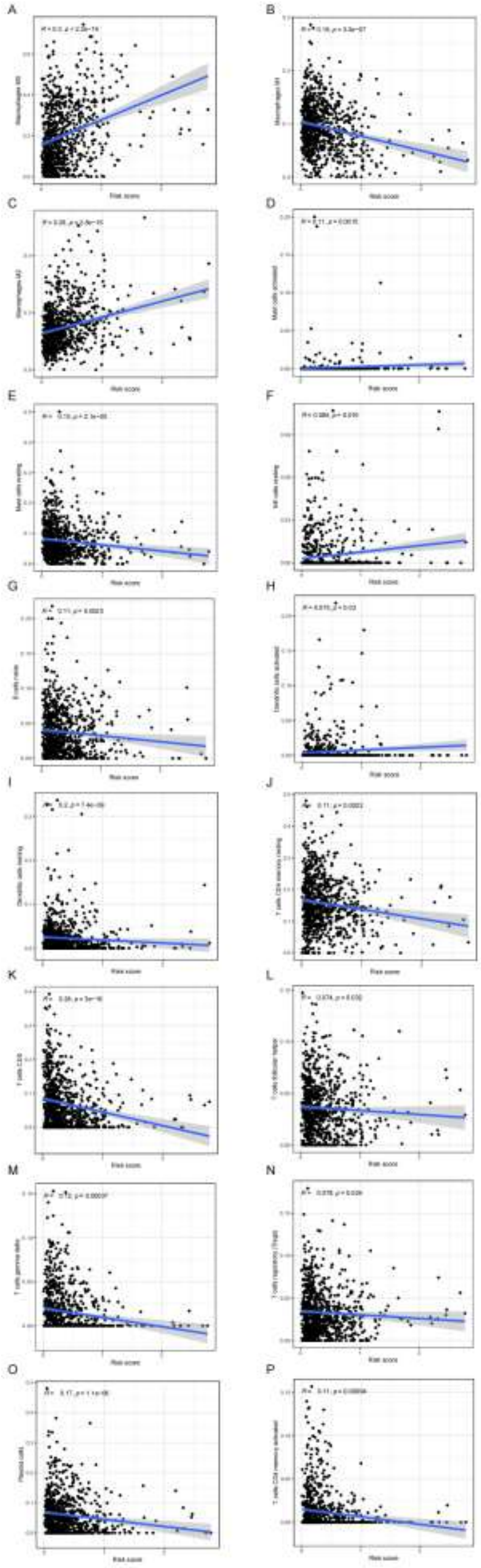
A
B
C
D
E
F
G
H
I
J
K
L
M
N
O
P
